# “Join the team” clinical research workforce development model: A new paradigm in healthcare career opportunities

**DOI:** 10.1017/cts.2024.577

**Published:** 2024-09-19

**Authors:** Kate Marusina, Olga Kishchenko, Angela Griffiths

**Affiliations:** Clinical and Translational Science Center, University of California Davis, Sacramento, CA, USA

**Keywords:** Clinical research, workforce development, training, model, internship

## Abstract

The University of California (UC) Davis Clinical and Translational Science Center has established the “Join the Team” model, a Clinical Research Coordinator workforce pipeline utilizing a community-based approach. The model has been extensively tested at UC Davis and demonstrated to generate a viable pathway for qualified candidates for employment in clinical research. The model combines the following elements: community outreach; professional training materials created by the Association for Clinical Research Professionals and adapted to the local environment; financial support to trainees to encourage ethnic and socioeconomic diversity; and internship/shadowing opportunities. The program is tailored for academic medical centers (AMCs) in recognition of administrative barriers specific to AMCs. UC Davis’s model can be replicated at other locations using information in this article, such as key program features and barriers faced and surmounted. We also discuss innovative theories for future program iterations.

## Introduction

The “Join the Team” initiative, a comprehensive science, technology, engineering, and mathematics-based workforce development pathway was developed by University of California (UC) Davis to address a perpetual shortage of qualified clinical research coordinators (CRCs). CRCs play a key role in the conduct of clinical trials; this profession demands a wide array of knowledge and skills to navigate ever-expanding therapeutic, regulatory, and logistical complexities. CRCs are in high demand with an annual 9.3% year-on-year growth in job postings, which does not keep pace with an annual 12.2% increase in clinical trials [1]. This biomedical research career path, which in principle does not require a 4-year degree, is largely unknown to the public [1].

Advarra National Trends survey (2021) demonstrated widespread concern about maintaining the current capacity of research studies in the absence of qualified clinical research staff [2]. Academic medical centers (AMCs) have suffered high CRC turnover rates due to uncertainty of job expectations and vague professional development opportunities, including a lack of role progression pathways, and disappointment with training. A 2022 Duke University exit survey demonstrated a significant trend towards leaving for higher-paid opportunities with Contract Research Organizations (CROs) and pharmaceutical sponsors, who also report a higher-than-average number of CRC job openings. Post-pandemic shortages of CRCs at AMCs are estimated at nearly 30% [3].

The current urgency in addressing CRC recruitment and retention is garnering national attention with multiple institutions reporting staff shortages. Staff shortages can have negative impacts on AMCs that may result in delaying or halting research activities, adversely affecting compliance and quality in the research, jeopardizing patient safety, or leading to further staff dissatisfaction, causing more turnover in a vicious cycle [4].

“Join the Team,” a comprehensive CRC workforce development initiative, uniquely addressed the CRC workforce shortage through the lens of increasing diversity and equity in biomedical research. Increasing the pool of racially and ethnically diverse staff who reflect the communities they serve can help address unconscious/implicit bias. This is particularly important to build trust among racialized groups, support more representative research, and thus improve generalizable outcomes, which ultimately is the systemic change we seek [5].

## Materials and methods

The “Join the Team” workforce development initiative is uniquely tailored to the needs of AMCs. The program is housed entirely at the UC Davis Clinical and Translational Science Center (CTSC) and is comprised of the following elements:

1. Community outreach;
2. Professional training materials created by the Association for Clinical Research Professionals (ACRP) (acrpnet.org) and adapted to the local environment;
3. Internship/shadowing opportunities; and
4. Financial support to trainees to encourage ethnic and socioeconomic diversity.

### Community outreach

One aim of the current program is to increase diversity in the clinical research workforce. Such initiatives can create a ripple effect in clinical research outcomes in general, as the lack of diversity in clinical research staff and physicians has been linked to lack of diversity in clinical research participants. Mistrust and misinformation about research continue to persist in our communities. Clinical Research Coordinators are at the critical interface between the community and science, and part of their job is to convey information about research goals and outcomes to the community. Moreover, when certain groups are not well represented in research, the data for safety and effectiveness of new medicines will be skewed in favor of overrepresented populations leading to even wider health disparities. Multiple national initiatives address physician diversity, but very few, if any, specifically address the diversity of clinical research staff [6,7].

The UC Davis CTSC previously established relationships with local community colleges that provide allied health professions programs, such as medical assistants, phlebotomists, or medical technicians. These partnerships prove reciprocally beneficial, capitalizing on the strengths of both the community colleges and UC Davis. In the local Sacramento community college district, students are 80% Black, Indigenous, and People of Color with considerable proportion of these identifying as first-generation college students, with more than 56% living below the poverty level. Initially, the Los Rios Community College District allied health faculty and student interest groups played a key role in shaping and disseminating information about clinical research coordination as a profession and specifically about this training opportunity, even though the Join the Team program has not been incorporated into the community college curriculum. The faculty also participated in the trainee selection and interview process, which included an extensive online application followed by a 15-minute Zoom interview with the selection panel. However, many applicants also found the program by searching the internet for training programs in healthcare. Applicants do not have to be active community college students to participate in the program.

As the program matured, the CTSC created specific targeting approaches for young adults from Sacramento’s Promise Zone neighborhoods, characterized by high poverty rates and home to populations underrepresented in the health research workforce. This targeted approach aims to improve diversity, equity, and inclusion in the clinical research profession, and ultimately, in clinical trials participation. Specifically, prospective program participants may be sought via collaborations with local community-based organizations, such as the Greater Sacramento Urban League (African-American), La Familia (Hispanic), or Asian Resources, Inc. (Asian).

### Training

The CRC Foundations Program is a 16-week session consisting of academic learning, workshops, field trips, and shadowing. Trainees participate in person two days per week. This unique program is built on Joint Task Force standards for CRC competencies (https://mrctcenter.org/clinical-trial-competency/). The academic learning portion is based on professional training materials developed by the ACRP (CRC Core Competency Foundations™ Training Program) [8]. In addition, we provide field trips with Compliance, Electronic Medical Records Research Team, Institutional Review Board, and many other important offices responsible for clinical research, as well as resume and interview workshops. The CTSC assists with applying for open positions. A mentor from senior clinical research staff is assigned to assist trainees during and postgraduation in adjusting to their new career in research. Currently, the program is conducted only in English.

The training materials cover the following essential topics:

- Knowledge, skills, and abilities to be a competent clinical research coordinator
- Different study designs
- General roles and responsibilities of the study personnel
- Essential documents and documentation
- Subject recruitment and retention process
- Investigational product management process
- Study communication
- Feasibility, site qualification, and site selection process
- Regulatory and ethical informed consent process
- Elements and purpose of good clinical practice
- CRC’s role in supporting monitoring process
- Types of adverse events (AEs) and processes for capturing, assessing, and reporting AEs

The first of the two in-person training days is largely dedicated to didactic learning, amounting to approximately 100 hours over the 16-week period. During the second training day, the trainees shadow CRCs from different clinical disciplines up to a maximum of 70 hours (see below).

The program culminates in the ACRP Clinical Research Knowledge Assessment™ as evidence of acquired competencies (80% required to pass). The sample schedule is shown in Table 1.

**Table 1.** Sample clinical research foundations curriculum

Day 1	Day 2
• Training based on the Association for Clinical Research Professionals (ACRP) Clinical Research Coordinator (CRC) Core Competency Foundations Training Program™	• Shadowing
	• Reflective learning posts
	• Weekly feedback surveys
	• Preparation for the next week field trips
	• Study hall
• Field Trips (options: Clinical Research Center, Radiology, Clinical Trials Office Finance/Regulatory/Feasibility Teams, RedCap, Institutional Review Board (IRB) Education, StudyPages, Investigational Drug Services, Compliance, Electronic Medical Records Research, Pathology)	

### Internship/shadowing

The CRC is an experiential profession, and it may take several years for a CRC to become fully proficient in this field. To ensure that the trainees have a better understanding of the CRC profession, we introduced multiple shadowing opportunities covering a variety of indications. The trainees have an ability to shadow experienced coordinators from a variety of clinical departments. The trainees highly value this shadowing experience as evidenced by the evaluation surveys. The initial trainees provided significant input into the curriculum and program structure, providing weekly feedback on course materials and shadowing opportunities. Feedback from the shadowing departments was also incorporated to alleviate the CRC burden and provide the best possible experience for both parties.

### Financial support

Join the Team targets individuals in underserved communities. The UC Davis CTSC has committed to funding participant stipends to support enrollment of trainees in financial need. The stipend was calculated as minimum wage (at that time, $15/hour in the state of California) multiplied by 170 training hours. This financial assistance became a vital support line for many of our trainees who need to take time off work to complete the training. To facilitate stipend administration, the trainees are onboarded with Medix Staffing Solutions, a nationwide healthcare and life sciences staffing agency (https://www.medixteam.com/). While there is a fee to use a staffing agency, onboarding with Medix provides an additional advantage of ensuring the graduates are fully vetted and immediately available as CRC contractors to UC Davis or anywhere in the country. The stipend is a key factor in attracting community to this training, with the long-term vision of interrupting the cycle of poverty, reshaping the social determinants of health, and ultimately building a more accessible and culturally responsive healthcare system.

### Implementation challenges

Launching a program of this scale and significance required a concerted effort of multiple individuals and organizations, such as the CTSC, Los Rios Community College District, Sierra Community College, the CTSC Health Equity Resources and Outreach program, the campus Diversity, Equity and Inclusion program, UC Davis HR, Medix, and others. The following notable barriers specific to academic health centers need to be taken into consideration when implementing community-based training programs.

#### Training pre-hire

The key aspect of the Join the Team program is the ability to train selected individuals pre-hire. This means that the selected participants are brought into the healthcare environment without having a status of an employee or a medical/undergraduate student. Our trainees did not fall into a “volunteer” or a “contractor” category as they could shadow but not interact with the UC Davis patients. To overcome this barrier, we identified a pertinent UC Davis policy that allowed community members to observe healthcare activities. We then sought permission from the Medical Executive Committee to utilize this policy in the context of our training. To onboard the first cohort, we worked with UC Davis Human Resources to provide background checks and medical clearances. Subsequently, Medix took over the onboarding activities (see below). Of note, we also leverage this program to cross-train existing UC Davis employees; these “internal cohorts” receive the same didactic training, but not shadowing. This allows the employees to train in under 100 hours, allowable by UC Davis cross-training guidelines. To date, 39 UC Davis employees have been cross-trained in the CRC role.

#### Stipends

Providing stipends to underserved trainees is a key element of the Join the Team program. However, we were not able to identify a pathway for generating stipends for the individuals not employed by UC Davis without creating individual contract agreements; our small administrative team was not able to handle such arrangements. The emerged solution capitalized on the existing UC Davis enterprise-wide contract with the Medix Staffing Solutions. Medix onboarded the trainees as UC Davis contractors, enabling seamless processing of stipends via electronic timecards. Eventually, Medix also assumed background checks and medical clearances. An additional advantage of Medix’s involvement was resume building and advice and the ability to immediately deploy the trainees in the workplace following the completion of the graduation requirements. While the program is geared toward generating clinical research workforce for UC Davis, it does not preclude the trainees from applying for jobs elsewhere. Moreover, we encourage the trainees to look for additional job opportunities. At least one graduate was hired by Sutter Healthcare, another significant local healthcare provider, as a clinical research coordinator.

#### Shadowing

The shadowing of an experienced CRC provides invaluable opportunity to learn about the profession, experience it in real life, and understand the prospective employers’ team dynamics and structure. However, the scheduling of shadowing opportunities proved to be a challenge due to multiple factors, such as CRC availability, clinical research visit schedules, and varied levels of engagement by clinical research teams and administration. The initial hesitation was dispelled after the first round of shadowing, as many CRCs found the mentoring/teaching experience to be deeply rewarding. The fact that most of our graduates were hired as career employees further strengthened the bond with the clinical research teams. To date, 14 clinical research teams have participated in the shadowing experience. At the time of the completion of this manuscript, the CTSC has entered into a tri-partite agreement with Medix and the Sutter Institute for Biomedical Research (SIMR), a research branch of Sutter Healthcare. This agreement enables our trainees to shadow SIMR CRCs at Sutter Healthcare clinics.

#### Cost

Join the Team is a high-touch program with personalized experience for a small group of individuals. The results speak for themselves with nearly 80% of all graduates employed in healthcare or clinical research careers. This approach comes at a significant cost, comprised of (1) license of the ACRP training materials; (2) trainer salary; (3) stipends; and (4) miscellaneous expenses (e.g., meals, parking, printing, etc.). While AMCs commonly create their own training materials, we continue using the ACRP package, which is professionally put together by the experts in the field. The materials are complete with well-developed PowerPoint presentations, case studies, worksheets, and eLearning. The ACRP materials are licensed on an annual basis [8]. The CTSC allocates efforts of a Program Manager (15%) and a Trainer (60%) to support program development and implementation.

## Results

The Join the Team commenced in Spring 2022. To date, 22 community trainees in four cohorts have successfully graduated from the program (see Table 2). Of these, 16 found jobs in clinical research, and one became a certified nursing assistant with University of California, Davis, Health. At the time of this publication, one graduate was still applying, and four decided not to pursue careers in healthcare. The trainees were predominantly female (18 out of 22). Eleven trainees self-identified as White, eight as Asian, two as Black, and one as Latino. All trainees had at least an Associate’s degree and more than half of the students had Bachelor of Science degrees. A vast majority had relevant healthcare experience as nurse aids, scribes, certified nursing assistants, medical assistants, or hospital volunteers. It is worth noting that we were not intentionally striving for diversified cohorts during the early years of the program. However, our latest collaboration with the Greater Sacramento Urban League (GSUL, see below) specifically emphasizes diverse socioeconomic and ethnic backgrounds. At the time of selection, all five GSUL trainees were unemployed, none identified as White/Caucasian, and none had healthcare experience or knowledge of medical terminology; two had only high school experience. The true test of the strength of our program will come from the success of this GSUL cohort.

**Table 2.** Demographic summary of the 22 graduates from the four community cohorts in the “Join the Team” program

**Gender**	
Females	18
Males	4
**Race/Ethnicity (self-identified)**	
White	11
Asian	8
Black	2
LatinX	1
**Highest degree before entering the Foundations Program**	
Associates (Science)	7
Associates (Art)	1
Bachelor (Science)	13 (includes two foreign graduates)
Bachelor (Art)	1
**Career postgraduation**	
% obtained a job in healthcare (clinical research or other)	17(77%)
% decided not to pursue career in research or health or still looking	5 (22%)

### Reflections

The Join the Team program garnered national recognition with the prestigious award from the Society for Clinical Research Sites. Our program stood up among others due to its unique aspects listed above and its ability to become a blueprint for other academic health centers or private sites. While we intentionally keep the number of trainees small, the scalability of the program comes from implementation of the blueprint in other locations. Notably, the CTSC has received the Californians For All workforce development grant to expand the training to individuals residing in specific zip codes of Sacramento. This expansion will be undertaken in collaboration with the Greater Sacramento Urban League to facilitate the enrollment and provide wrap-around social support to trainees. The grant also creates an opportunity for about 600 hours of paid internship to provide deeper engagement into the profession and increase the likelihood of securing a career position. The Urban League is a national civil rights and workforce development organization with chapters in multiple locations throughout the country. The success of the UC Davis implementation will pave the way for other CTSA-Urban league workforce collaborations.
